# Differential Modulation of the Central and Peripheral Monoaminergic Neurochemicals by Deprenyl in Zebrafish Larvae

**DOI:** 10.3390/toxics9060116

**Published:** 2021-05-23

**Authors:** Marina Bellot, Helena Bartolomé, Melissa Faria, Cristian Gómez-Canela, Demetrio Raldúa

**Affiliations:** 1 Department of Analytical Chemistry and Applied (Chromatography Section), School of Engineering, Institut Químic de Sarrià-Universitat Ramon Llull, Via Augusta 390, 08017 Barcelona, Spain; marina.bellot@iqs.url.edu (M.B.); helenabartolomea@iqs.url.edu (H.B.); 2 Institute for Environmental Assessment and Water Research (IDAEA-CSIC), Jordi Girona, 18, 08034 Barcelona, Spain; milfculb@gmail.com

**Keywords:** zebrafish larvae, monoaminergic neurochemicals, central nervous system, peripheral nervous system

## Abstract

Zebrafish embryos and larvae are vertebrate models increasingly used in translational neuroscience research. Behavioral impairment induced by the exposure to neuroactive or neurotoxic compounds is commonly linked to changes in modulatory neurotransmitters in the brain. Although different analytical methods for determining monoaminergic neurochemicals in zebrafish larvae have been developed, these methods have been used only on whole larvae, as the dissection of the brain of hundreds of larvae is not feasible. This raises a key question: Are the changes in the monoaminergic profile of the whole larvae predictive of the changes in the brain? In this study, the levels of ten monoaminergic neurotransmitters were determined in the head, trunk, and the whole body of zebrafish larvae in a control group and in those treated for 24 h with 5 M deprenyl, a prototypic monoamine-oxidase B inhibitor, eight days post-fertilization. In control larvae, most of the monoaminergic neurochemicals were found at higher levels in the head than in the trunk. Significant changes were found in the distribution of some neurochemicals after deprenyl-treatment, with serotonin and norepinephrine increasing in both the head and the trunk, whereas dopamine, L-DOPA, and homovanillic acid levels were only modulated in the head. In fact, the highly significant increase in dopamine levels observed in the head after deprenyl-treatment was not detected in the whole-body analysis. These results indicate that the analysis of neurotransmitters in the zebrafish larvae whole-body should not be used as a general surrogate of the brain.

## 1. Introduction

Neurotransmission, the basis of neuronal communication, is critical for a normal neural function [1]. Monoamine neurotransmitters, especially the catecholamines dopamine and norepinephrine as well as the tryptamine serotonin, are major modulatory mechanisms in the vertebrate brain related with motor control, emotion, stress, and cognition [1,2]. Different neuroactive compounds, including illicit drugs, pharmaceuticals, and environmental pollutants, are able to impair the levels of monoaminergic neurotransmitters in the brain through the inhibition of their synthesis, re-uptake, and metabolism [3,4,5]. Altered monoaminergic neurotransmission in the brain has been linked to several neurological disorders such as Alzheimer’s and Parkinson’s diseases, neuropsychiatric disorders, and depression [6]. However, the modulatory role of monoaminergic neurotransmitters is not restricted to the brain or the central nervous system (CNS), as these neurochemicals are also involved in important signaling functions throughout our body. For instance, dopamine and norepinephrine produced by enteric neurons are involved in gut motility and secretory reflexes [7]. Serotonin is particularly abundant in the intestine (about 90% of the serotonin in the body), where it is produced largely by enterochromaffin cells (EC) but also by enteric neurons; it is involved in gut motility and enteric neuron development [7,8]. In the enteric nervous system (ENS), both catecholamines and serotonin are involved in immunomodulation, and changes in the levels of monoaminergic neurotransmitters in the intestine have been associated with inflammatory bowel disease in humans [9]. However, the monoaminergic neurotransmitters located in the gastrointestinal tract are not only produced by the EC and enteric neurons but also by commensal gut microorganisms; they may play a role as signaling molecules, mediating the function of the “microbiota–gut–brain” axis [10].

Zebrafish is an animal model widely used for studying vertebrate development and human diseases, including neurobehavioral diseases [11]. In fact, zebrafish has emerged as a new and powerful model species in translational neuroscience because it exhibits an overall nervous system organization and neurotransmitter systems similar to humans [12]. The availability of comprehensive behavioral repertoires for larval and adult zebrafish further enhances the utility of this model species for translational neuroscience research [13]. As a result, the zebrafish model is increasingly being used to analyze the mechanisms and effects of the dysregulation of neurotransmitters homeostasis on different behaviors. The use of zebrafish early larvae as an experimental model has several advantages compared to models using adults or embryos. The size of early larvae (about 3.5 mm) is still small enough for performing HTS, and their CNS is already quite well developed, including the main neurotransmitter systems [14]. Different studies have determined the effect of different environmental pollutants and drugs on the profile of neurotransmitters in embryos and larvae of this species [15,16,17,18,19]. Because of the small size of the early zebrafish larvae, it is extremely difficult to perform a clean dissection of the brain in a time short enough for avoiding degradation of neurochemicals. As a result, in most of the studies the neurotransmitter profile has been determined in the whole body of the larvae and only a few studies have analyzed the neurotransmitter content in the head of larvae [20] or juvenile [21,22]. Currently, the information about the central and peripheral distribution of monoaminergic neurochemicals in zebrafish larvae is scarce. Moreover, information about a potential differential regulation by neuroactive chemicals of monoaminergic neurochemicals between the central and peripheral system is missing. In these conditions, it is hard to know if the changes observed in the neurotransmitter profile obtained from the whole body of the larvae are predictive of the changes in their brains. This is an important problem when looking for potential relationships between behavioral changes and the altered profile of brain monoaminergic neurotransmitters. Therefore, there is an urgent need to increase our current knowledge on the distribution of neurotransmitters in the zebrafish larval body, as well as in determining the predictive value of using the neurotransmitter profile in the whole body for identifying changes occurring in the brain.

In this manuscript we analyzed the distribution of 10 monoaminergic neurochemicals in the head, trunk, and whole body of wild-type zebrafish larvae control and those exposed for 24 h to deprenyl, a prototypic monoamine-oxidase (MAO) inhibitor, 8 days post-fertilization (dpf). First, the monoaminergic profiles in head and trunk were compared in both control and treated larvae. Then, the monoaminergic profiles found in the head and whole body of the exposed larvae were compared.

## 2. Material and Methods

### 2.1. Chemicals and Reagents

Standards of L-tyrosine (Tyr), levodopa (L-DOPA), dopamine hydrochloride (DA), dihydroxyphenylacetic acid (DOPAC), homovanilic acid (HVA), L-tryptophan (Trp), and serotonin hydrochloride (5-HT) were obtained from Sigma-Aldrich (St. Louis, MO, USA). The 5-hydroxyindoleacetic acid (5-HIAA) was obtained from Toronto Research Chemicals (TRC, Toronto, ON, Canada), 3-methoxytyramine hydrochloride (3-MT) was purchased from Merck (Darmstadt, Germany), and norepinephrine (NE) was provided by Tocris Bioscience (Ellisville, MO, USA). Stock solutions of all the neurotransmitters were prepared at 1 µg µL^−1^ in ultra-pure water, MeOH, or DMSO. In addition, labeled standards L-tryptophan-1-13C, 5-hydroxyindole-3-acetic acid-d5, L-DOPA-2,5,6-d3, and L-Tyrosine-13C9,15N were all supplied by Toronto Research Chemicals (TRC, Toronto, ON, Canada). A mix solution of all labeled standards (internal standard mixture, ISM) was prepared in extractant solvent (1 ng µL^−1^). These standards were kept in amber vials at −20 °C to prevent degradation. Acetonitrile (ACN) of HPLC LC-MS grade was purchased from VWR Chemicals (Leuven, Belgium) and ultra-pure water was obtained through Millipore Milli-Q purification system (Millipore, Bedford, MA, USA). Formic acid (FA) was supplied by Fischer Scientific (Loughborough, UK) and ammonium formate by Sigma-Aldrich (St. Louis, MO, USA).

### 2.2. Fish Husbandry and Larvae Production

Adult wild-type zebrafish were purchased from EXOPET (Madrid, Spain) and maintained in fish water (reverse-osmosis purified water containing 90 μg/mL of Instant Ocean (Aquarium Systems, Sarrebourg, France) and 0.58 mM CaSO_4_·2H_2_O) at 28 ± 1 °C in the Research and Development Center of the Spanish Research Council (CID-CSIC) facilities under standard conditions. Embryos were obtained by natural breeding with a 5:3, female/male ratio per tank. Embryos deposited in the bottom of the breeding tank were collected and maintained in 500 mL glass containers at 1 embryo/mL density in fish water at 28.5 °C on a 12 light/12 dark photoperiod. Larvae were not fed before or during the experimental period (from 7 to 8 days post fertilization (dpf)). All procedures were approved by the Institutional Animal Care and Use Committees at the CID-CSIC and conducted in accordance with the institutional guidelines under a license from the local government (agreement number 9027).

### 2.3. Experimental Protocol

The chemical selected for this study was deprenyl (CAS14611-52-0), of certified laboratory high quality grade purchased from Sigma-Aldrich (St. Louis, MO, USA). The stock solution, 5 mM deprenyl, was prepared in DMSO and then diluted to 5 µM deprenyl (working solution) in fish water. The deprenyl concentration used in this study, 5 μM, was selected from our previous study, Faria et al. (2019) [23]. In that study, 5 μM was the maximum tolerated concentration of deprenyl for systemic toxicity. Moreover, in that study we found that when 7 dpf larvae were exposed for 24 h to 5 μM deprenyl, they exhibited a significant increase in habituation to vibrational stimuli, with a special reduction in the escape responses to the first 8–10 tapping stimuli. Vehicle controls with 0.1% DMSO were used in this study, as this DMSO concentration has been reported to be safe and is widely used to screen libraries of small chemicals in zebrafish [24,25]. Exposures were conducted in 48-well microplates with 1 larva per well and 1 mL of working solution. After 24 h of exposure (7 to 8 dpf) control and treated larvae were collected, euthanized by freezing, and stored at −80 °C until neurochemical analyses. The exposure window was chosen because the central nervous system in 7 dpf zebrafish larvae is quite well developed so the observed effects will be mainly related to neurotoxicity instead of developmental neurotoxicity. A longer exposure was rejected to avoid having to feed the larvae and therefore insert a new variable into the experiment. For head and trunk analysis, larvae were euthanized by chilling prior to decapitation using fine iridectomy scissors and No. 5 watchmaker forceps [26]. The head was sectioned with the larvae positions on the lateral side, immediately caudal to the otic vesicle and cranial to the anterior intestine (see Supplementary Figure S1). All the exposures were performed at 28.5 °C (POL-EKO APARATURA Climatic chamber KK350, Poland) with a 12L:12D photoperiod. Samples were collected from two trials conducted in different days and with different larvae batches.

### 2.4. Extraction and Analysis of Neurochemicals

Monoaminergic neurochemicals were extracted from 8 pools of 5 whole-larvae, 8 pools of 15 heads, and 8 pools of 15 trunks following an extraction procedure adapted from Mayol-Cabré et al. (2020) [27]. The extraction process was based on the use of a solvent of polarity similar enough to the neurotransmitters to be able to extract them from the sample. First, the samples with the extractant solvent were homogenized using stainless steel beads in a mill homogenizer (TissueLyser LT, Quiagen, Hilden, Germany). Then, the resulting supernatant was centrifuged and filtered and introduced into a chromatographic vial. The analysis was performed by ultra-high-performance liquid chromatography (Acquity UPLCH-Class Waters, Milford, MA, USA) coupled to a triple quadrupole mass spectrometer (Xevo, TQS micro, Waters, Milford, MA, USA) equipped with an electrospray ionization source (ESI). Total protein of the samples was measured by the Bradford method [28] using bovine serum albumin (BSA) as the standard. Results were normalized by two different methods, by larva and by protein content. Additional details on the extraction and analysis of neurotransmitters are provided in the Supplementary Methods and Supplementary Table S1.

### 2.5. Statistical Analysis

After checking the 480 results obtained from the neurochemical analysis, 3 outliers were identified for data normalized by protein and 4 outliers for data normalized by larva (see Supplementary Dataset 1). Outliers were excluded from the statistical analysis. Data were analyzed with IBM SPSS v25 (Statistical Package 2010, Chicago, IL, USA). Normality was assessed using the Kolmogorov–Smirnov and Shapiro–Wilk tests. The Student’s *t*-test was used to test for differences between normally distributed groups. Data are presented as by the mean ±SE. Significance was set at *p*  <  0.05.

## 3. Results

### 3.1. Distribution of Monoaminergic Neurochemicals between the Head and the Trunk in Control and Deprenyl-Treated Larva

**Figure 1:**
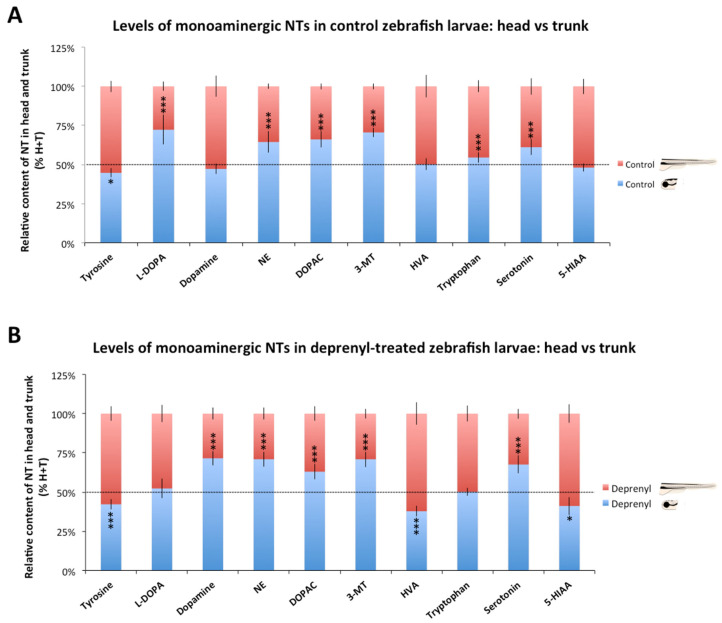
Levels of monoaminergic neurochemicals in the head and trunk of zebrafish larvae. (**A**) Distribution in the head and the trunk of control larvae 8 days post-fertilization (dpf); (**B**) Distribution in the head and the trunk of 8 dpf larvae exposed for 24 h to 5 µM deprenyl, a monoamine-oxidase inhibitor, in the water. Data were normalized by protein content (n = 8 pools) * *p* < 0.05, *** *p* < 0.001; Student’s *t*-test; Data from 2 independent experiments.

Figure 1A shows the profile of the 10 selected monoaminergic neurochemicals in the head and trunk of control larvae, using data normalized by protein content (Supplementary Table S2 contains information on the protein content in whole body, heads, and trunks of control and deprenyl-treated larvae pools). Whereas L-DOPA (*p* = 4.2 × 10^−9^), NE (*p* = 1.77 × 10^−6^), DOPAC (*p* = 2.6 × 10^−9^), 3-MT (*p* = 1.12 × 10^−9^), tryptophan (*p* = 2.58 × 10^−5^), and serotonin (*p* = 1.2 × 10^−5^) exhibited significantly higher levels in the head, only tyrosine exhibited significantly higher levels than the trunk (*p* = 0.0013). Dopamine (*p* = 0.56), HVA (*p* = 0.52), and 5-HIAA (*p* = 0.43) exhibited similar levels in the head and trunk. When data were normalized by larva (Supplementary Figure S2A), all the selected neurochemicals exhibited higher levels in the head, with levels ranging from 56.1% of tyrosine to 79.6% of L-DOPA. The five most abundant neurochemicals in the head of control larvae were similar with both methods of normalization: L-DOPA > 3-MT > DOPAC > NE > serotonin.

**Figure 2:**
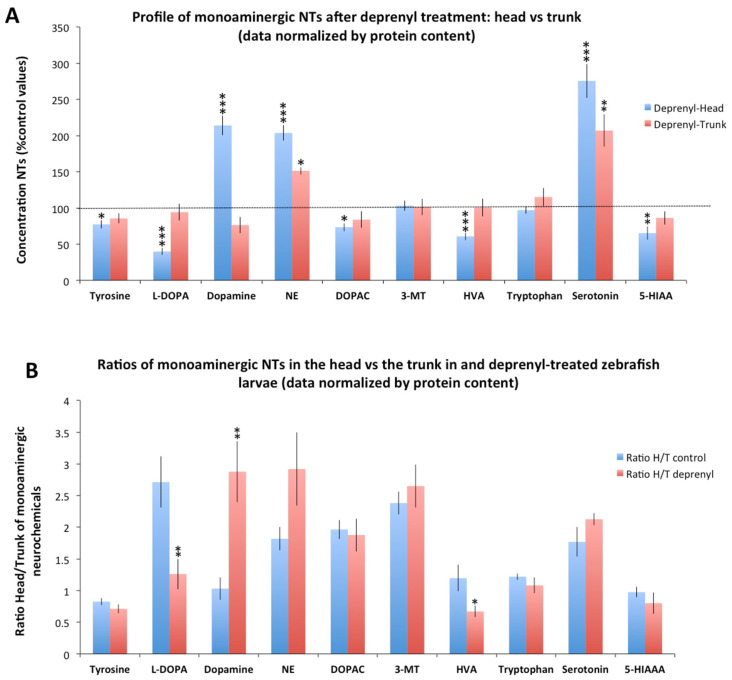
Distribution of monoaminergic neurochemicals between the head and trunk of zebrafish larvae exposed to 5 µM deprenyl for 24 h. (**A**) Concentration of monoaminergic neurochemicals in the head and trunk of larvae exposed to deprenyl, expressed as a percentage of the control values; (**B**) Ratio of head/trunk for the ten selected monoaminergic neurochemicals in control and deprenyl-treated zebrafish larvae. Data was normalized by protein content. Student’s *t*-test (n = 8 pools); * *p* < 0.05, ** *p* < 0.01, *** *p* < 0.001; Data from 2 independent experiments.

Significant changes were found in the distribution of some neurochemicals between the head and the trunk in deprenyl-treated larvae. In contrast to the distribution observed in control larvae, when data normalized by protein were used, the head of the treated larvae exhibited higher levels of dopamine (*p* = 1.23 × 10^−7^) and lower levels of HVA (*p* = 0.0005) and 5-HIAA (*p* = 0.025) than those in the trunk (Figure 1B). Similar results were obtained when the data were normalized by larva (Supplementary Figure S2B). Then, the specific changes in the monoaminergic profile occurring in the head and the trunk were evaluated. As Figure 2A shows, deprenyl significantly increased serotonin and NE levels in both head and trunk. Interestingly, dopamine levels were modified only in the head, exhibiting a high and significant increase in response to deprenyl. Finally, levels of tyrosine, L-DOPA, DOPAC, HVA, and 5-HIAA decreased only in the head of the deprenyl-treated larvae.

To explore in depth the distribution on neurochemicals in the head and trunk of control and deprenyl-treated larvae, the head/trunk (H/T) ratios were calculated (Figure 2B). If deprenyl-treatment had a similar effect on the central and peripheral monoaminergic system, the H/T ratio in the treated larvae should be similar to that in the controls. However, H/T ratios for L-DOPA, dopamine, and HVA in deprenyl-treated larvae were significantly different to those from controls with both normalization methods (by protein content: L-DOPA (*p* = 0.008), dopamine (*p* = 0.003), and HVA (*p* = 0.037); by larva: L-DOPA (*p* = 0.002), dopamine (*p* = 0.007), and HVA (*p* = 0.013)), a result indicating a differential effect of deprenyl in the head and trunk of the larvae.

### 3.2. Differences in the Neurochemical Profile Obtained from the Head and the Whole Body of the Larvae after Deprenyl-Treatment 

**Figure 3:**
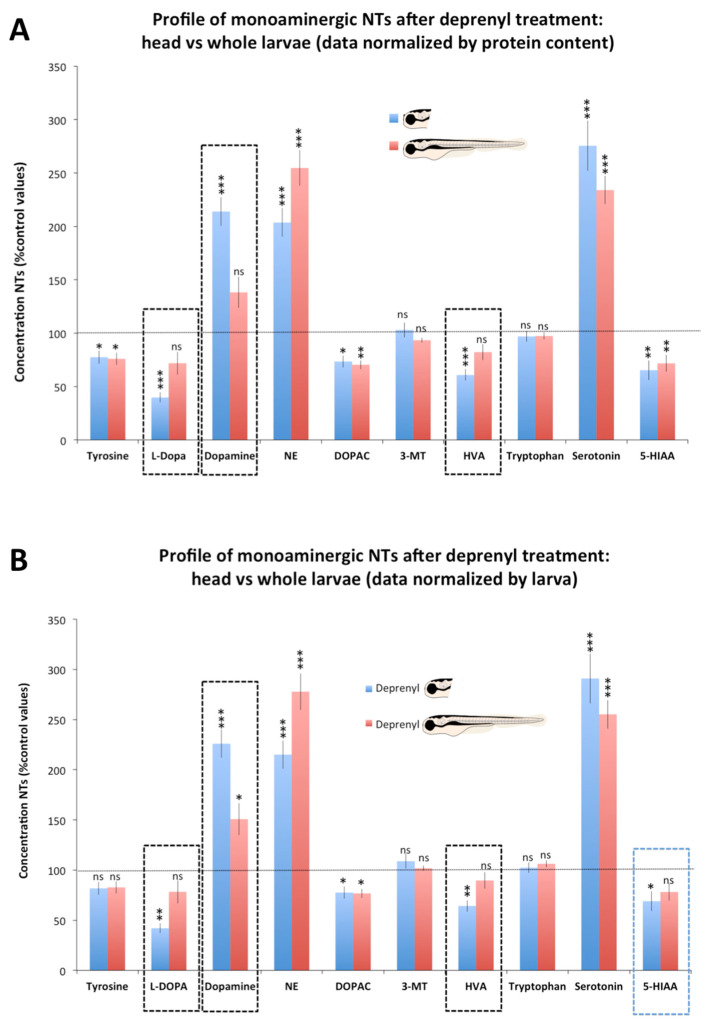
Profile of monoaminergic neurochemicals after deprenyl treatment: head vs. whole body. Concentrations are presented as percentage of the control values. (**A**) Data normalized by protein content; (**B**) Data normalized by larva. Student’s *t*-test (n = 8 pools); ns: non-significant, * *p* < 0.05, ** *p* < 0.01, *** *p* < 0.001; Data from 2 independent experiments.

Figure 3 and Supplementary Figure S3 compare the level of each neurochemical in the head and whole body of larvae exposed to deprenyl, as a percentage of their respective control values. A significant decrease in L-DOPA (*p* = 0.0007 for data normalized by protein and by larva) and HVA (*p* = 0.0007 and *p* = 0.0016 for data normalized by protein by larva, respectively) was found in the head but not in the whole body of deprenyl-treated larvae compared with their respective controls. Moreover, in spite to the highly significant increase in dopamine found in the heads of the larvae exposed to deprenyl (*p* = 2.56 × 10^−6^ and *p* = 1.34 × 10^−6^ for data normalized by protein and by larva, respectively), when the whole body was analyzed, only mild increases (*p* = 0.048 for data normalized by larva) or no differences with the control (*p* = 0.112 for data normalized by protein content) were found.

### 3.3. Variability of the Neurochemical Data Obtained from Heads and Whole Bodies

**Figure 4:**
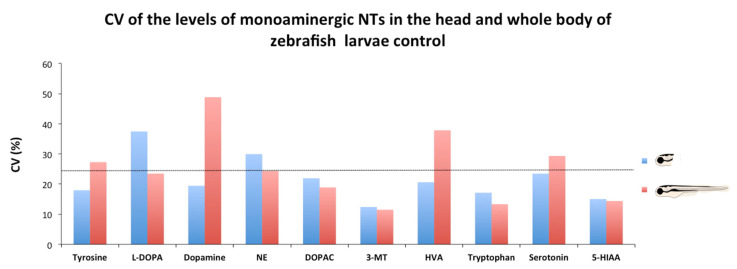
Analysis of the variability of the determination of neurochemicals in the head and the whole body of control larvae. Coefficient of variation was used for determining the variability of the concentrations of the monoaminergic neurochemicals determined in the head and the whole body.

Sectioning heads of zebrafish larvae is an easy and fast process, but also a potential source of variability of the data, as it is difficult to section all the heads exactly at the same level. Therefore, the coefficients of variation (CV) of the neurotransmitter data obtained from heads and the whole body were compared. As Figure 4 shows the data variability in the head was lower than that in the whole body for tyrosine, dopamine, HVA, and serotonin. In contrast, data variability was lower in the whole larvae for L-DOPA, NE, DOPAC, and tryptophan. Finally, variability for 3-MT and 5-HIAA was very similar in heads and the whole body. Only two of the ten neurochemicals analyzed (L-DOPA and NE) exhibited a CV above 25% in the head, whereas four of them (tyrosine, dopamine, HVA, and serotonin) exhibited a CV above 25% in the whole-body samples.

## 4. Discussion

Whereas the level of monoaminergic neurotransmitters in specific areas of the brain modulates behaviors such as mood, aggressiveness, or fear, the presence of these neurochemicals is not restricted to the CNS, as they are also peripherally distributed. It is not clear if the exposure to neuroactive chemicals targeting the synthesis, re-uptake, or metabolism of these neurotransmitters leads to similar effects on the central and peripheral pools of these chemical messengers. In this study, the distribution of the monoaminergic neurochemicals between the head and trunk in control and deprenyl-treated zebrafish larvae was analyzed. The exposure conditions used in this work, 5 µM deprenyl from 7 to 8 dpf, lead to the total abolition of the MAO activity and impairment of different behaviors (to be published elsewhere); therefore, changes in the serotonergic and/or dopaminergic neurochemicals in the brain should be expected. In that case, why did we analyze the heads instead of brains? As a result of the small size of 8 dpf zebrafish larvae, a clean dissection of the brain is only possible with specific transgenic lines expressing fluorescent proteins in the brain [29]. In any case, even if possible, it is a complex and time-consuming process, which results in a high risk of degradation of some of the neurochemicals. In contrast, to section a zebrafish larva into head and trunk is a fast and easy process preserving the integrity of the neurochemical pool of the brain. Although the head contains more than just the brain, for example, some non-CNS tissue such as skin, cartilages, muscle, and gills [26], it is highly enriched in brain tissue. Therefore, the neurochemical profile obtained from heads should provide a good approximation of the situation in the brain, where the neural circuits involved in the different behaviors are located. The content of monoaminergic neurochemicals in the head of zebrafish early larvae was previously determined by Chen et al. (2016) [20], but in that case eyes were removed during the sampling of the heads. Despite its peripheral location, the retina is part of the central nervous system. Therefore, it makes sense to include the eyes when the neurotransmitter profile of the CNS needs to be analyzed. However, if the aim of the analysis is to analyze changes in specific brain areas or to link behavioral changes with changes in neurotransmitter profile, then eyes should be removed.

In this study, when the distribution of the neurochemicals between the head and the trunk was analyzed, the head presented significantly higher levels of L-DOPA, NE, DOPAC, 3-MT, tryptophan, and serotonin than those found in the trunk of control larvae using both normalization systems. Whereas intestines store 90–95% of the serotonin of the body in mammals [30], in this study, only 27% (data normalized by larva) of the serotonin was stored in the trunk of the control larvae. The limited content of serotonin in the trunk of the larvae might be related with the restricted distribution of serotonin-containing EC cells in the distal intestinal epithelium [31]. Interestingly, serotonin levels in the intestine of zebrafish larvae seem be modulated by drugs, including MAO inhibitors, serotonin-re-uptake inhibitors (SSRI), and tryptophan hydroxylase inhibitors [30]. Therefore, drugs commonly used to regulate serotonin levels in the human brain have a similar effect on the serotonin levels at the intestine of zebrafish larvae. In fact, when in this study the effect of deprenyl, a prototypic MAO inhibitor, was assessed in the head and trunk of the larvae, a similar increase in serotonin levels was found in both parts of the body (Figure 1 and Figure 2). Interestingly, the effect of deprenyl on the whole-body serotonin was very similar to the effect in the head (Figure 3), a result supporting the use of the whole-body larva as a surrogate of the head to determine changes in serotonin levels. For dopamine, another relevant monoaminergic neurotransmitter, the scenario was very different. Whereas in control larvae this neurotransmitter was equally distributed between head and trunk, deprenyl exposure led to a significant increase in dopamine levels only in the head (Figure 1 and Figure 2). As a result, when the dopamine levels were analyzed in the whole larvae after deprenyl exposure, no differences were found with respect to the control, in spite of the highly significant increase found in the heads. This fact probably explains the findings reported by Sallinen et al. (2009), in which the analysis of serotonin and dopamine in the whole body of 5 dpf zebrafish eleutheroembryos exposed to 100 µM deprenyl from the early development resulted in a significant increase in the serotonin levels but no effect on the dopamine levels. Differences in the affinity and turnover number of zebrafish MAO (zMAO) toward dopamine and serotonin might also contribute to the observed differences in the effects of deprenyl on these two neurotransmitters. However, the reports on the suitability of serotonin and dopamine as zMAO substrates are contradictory. Anichtchik et al. (2006) [32] reported a much higher affinity of zMAO for serotonin than for dopamine, but the same authors suggested that dopamine results might be affected by the presence of ascorbic acid in the incubation solution. More recently, Aldeco et al. (2011) [33] found that zMAO oxidizes dopamine with a turnover number similar to that of serotonin (Kcat values of 242 min^−1^ and 187 min^−1^, respectively). In fact, the increase in the dopamine and serotonin levels found in the head of deprenyl-exposed larvae in this study were quite similar (214 vs. 275% of the control values, respectively), supporting the similar kinetic parameters of zMAO for these substrates reported in the Aldeco et al. (2011) study [33].

However, the differential modulation of monoaminergic neurochemicals after deprenyl-treatment between the head and the whole body was not restricted to dopamine. Similarly, the analysis of the monoaminergic profile in the whole larvae missed the decrease in the L-DOPA and HVA detected when the heads were analyzed (Figure 3).

Another important issue we considered in this manuscript is the data normalization process. Since it is not feasible to weigh larvae pools, data were normalized by protein content and by number of larvae, though the latter can obscure results due to collection errors and differential larvae sizes. 

## 5. Conclusions

The results presented in this manuscript clearly indicate that the neurochemical profile of the whole zebrafish larvae should not be used as a general surrogate of the profile in the brain. This is an important fact to be considered in future studies aiming to link behavioral impairment with neurochemical changes in the brain of zebrafish after exposure to neuroactive and/or neurotoxic chemicals.

## Data Availability

All data are contained in the manuscript and the Supplementary Material and Supplementary Datasheet 1 files.

## Supplementary Materials

The following are available online at https://www.mdpi.com/article/10.3390/toxics9060116/s1. Supplementary Methods: Extraction and Analysis of Neurochemicals; Figure S1: Supplementary Figure S1. Level of the section of the head and trunk in 8 days post-fertilization zebrafish larvae; Figure S2: Levels of ten monoaminergic neurochemicals in the head and trunk of zebrafish larvae (data normalized by larva); Figure S3: Distribution of ten monoaminergic neurochemicals between head and trunk of zebrafish larvae exposed to 5 μM deprenyl for 24 h (data normalized by larva); Supplementary Table S1: Quality parameters obtained by LC-MS/MS for the 10 monoaminergic neurochemicals; Supplementary Table S2: Protein content in whole body, heads, and trunks of control and deprenyl-treated 8 days post-fertilization zebrafish larvae; Supplementary Dataset 1: Data on neurochemicals levels in the head, trunk, and whole body of larvae control and those exposed to 5 μM deprenyl for 24 h, normalized by protein content and larva.
