# Supplementary material for: Differential Modulation of the Central and Peripheral Monoaminergic Neurochemicals by Deprenyl in Zebrafish Larvae
Source: Toxics. 2021 May 23;9(6):116. doi: 10.3390/toxics9060116 (PMC8224676; doi:10.3390/toxics9060116)
Supplement: Supplementary file 1 [file toxics-09-00116-s001.zip › toxics-1208916- SI.pdf]

Article

Differential Modulation of the Central and Peripheral Monoaminergic Neurochemicals by Deprenyl in Zebrafish Larvae: Supplementary Material

1.1Marina Bellot [1], Helena Bartolomé [1], Melissa Faria [2], Cristian Gómez-Canela [1,*] and Demetrio Raldúa [2,*]

[1] Affiliation 1; e-mail@e-mail.com
[2] Affiliation 2; e-mail@e-mail.com
* Correspondence: e-mail@e-mail.com; Tel.: (optional; include country code; if there are multiple corresponding authors, add author initials)

Abstract: A single paragraph of about 200 words maximum. For research articles, abstracts should give a pertinent overview of the work. We strongly encourage authors to use the following style of structured abstracts, but without headings: (1) Background: Place the question addressed in a broad context and highlight the purpose of the study; (2) Methods: briefly describe the main methods or treatments applied; (3) Results: summarize the article's main findings; (4) Conclusions: indicate the main conclusions or interpretations. The abstract should be an objective representation of the article and it must not contain results that are not presented and substantiated in the main text and should not exaggerate the main conclusions.

Keywords: keyword 1; keyword 2; keyword 3 (List three to ten pertinent keywords specific to the article yet reasonably common within the subject discipline.)

Citation: Bellot, M.; Bartolomé, H.; Faria, M.; Gómez-Canela, C.; Raldúa, D. Differential Modulation of the Central and Peripheral Monoaminergic Neurochemicals by Deprenyl in Zebrafish Larvae. *Toxics* **2021**, *9*, 116. https://doi.org/10.3390/toxics9060116

Academic Editor: Ki-Tae Kim

Received: 19 April 2021
Accepted: 21 May 2021
Published: 25 May 2021

Publisher's Note: MDPI stays neutral with regard to jurisdictional claims in published maps and institutional affiliations.

Copyright: © 2021 by the authors. Licensee MDPI, Basel, Switzerland. This article is an open access article distributed under the terms and conditions of the Creative Commons Attribution (CC BY) license (http://creativecommons.org/licenses/by/4.0/).

1. Supplementary Methods

1.1. Extraction and Analysis of Neurochemicals

First, 300 µL of the cold extractant solvent (ACN/H_2O 90:10 + 1% FA) were added to samples. Pools were spiked with an isotope labeled solution (ISM) used as internal standard. The concentration was assessed checking the response of each neurochemical. Then, three stainless steel beads (3 mm diameter) were placed in each sample and were homogenized in a mill homogenizer (TissueLyser LT, Quiagen, Hilden, Germany) at 50 osc/sec during 90 sec. Subsequently, the supernatants were centrifuged at 4 °C for 20 min at 13,000 rpm. Finally, the samples were filtered using 0.22 µm PTFE filters (DISMIC -13 JP, Advantec®) and kept at -20 °C until the analysis.

1.2. Quality assurance

The suitability of the method for all the analytes was studied in the range from 0.005 to 2.5 ng μL^{-1}. The ISM was used as extraction and analytical internal standard. To study the recovery of the extraction method five replicates of head, trunk and whole larvae pools were

spiked at 50 ng with the native neurochemical standard mixture and ISM (QC). Instrumental detection limits (IDLs) were determined using the lowest concentrated standard (0.005 ng μL^{-1}) that yielded a S/N ratio equal to 3, while method detection limits (MDLs) were calculated using QCs. Intra-day precision was assessed by three consecutive injections of 1 ng μL^{-1} standard solution and inter-day precision was evaluated by measuring the same standard solution for four different days. Moreover, matrix effect (ME) was determined by comparing the peak area of each NT from the QCs with the peak area of the analyte from the standard solution used in calibration curve. This parameter is used to determine if a suppression or enhancement of the analytes was occurring.

2. Results and discussion

2.1. Quality parameters

Great correlation coefficients (r2) were obtained over 0.99 for most analytes in a range from 0.005 to 2.5 ng μL^{-1}. Moreover, IDLs were ranged from 1.00 pg (3-MT) to 33.3 pg (HVA). Furthermore, intra-day precision was ranged from 2.1% to 6.3% and inter-day precision values were from 4.3% to 10.8%. MDLs, recoveries and ME were calculated for heads, trunks, and whole larvae samples. Recoveries were all ranged between 65 and 135%, except for Trp (57%) and NE (140%) in head (57%), 3-MT (138%) and Tyr (140%) in trunk. For heads, the MDLs varied from 0.3 (3-MT) to 44.3 (HVA) ng head^{-1}and, the matrix effect suggested signal suppression (below 70%) for NE and Tyr, while suggested signal enhancement (above 130%) for 3-MT, Trp and DA. In the case of trunks, MDLs were ranged from 0.1 (3-MT) to 34.9 (HVA) ng trunk^{-1} and, the matrix effect suggested signal enhancement for 3-MT and Trp. Finally, MDLs of whole larvae samples varied from to 2.6 (5-HT) to 54.5 (HVA) ng larvae^{-1}. The matrix effect suggested signal suppression for 3-MT, DOPAC and LD, while suggested signal enhancement for Trp. All the quality parameters above mentioned are summarized in Supplementary Table T1.

3. Supplementary Figures

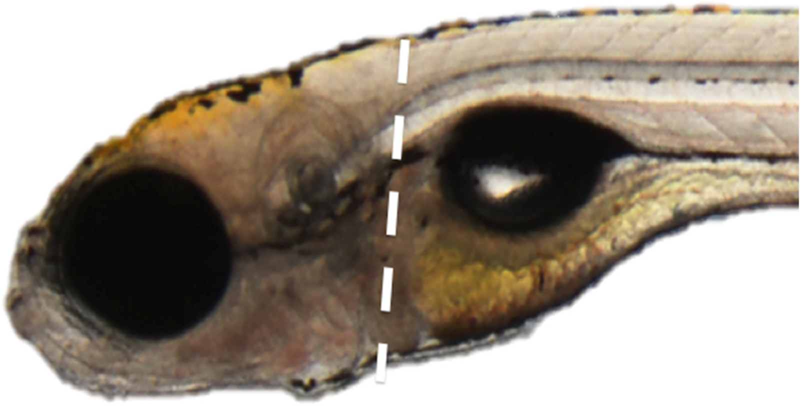

Figure S1. Level of the section of the head and trunk in 8 days post-fertilization zebrafish larva

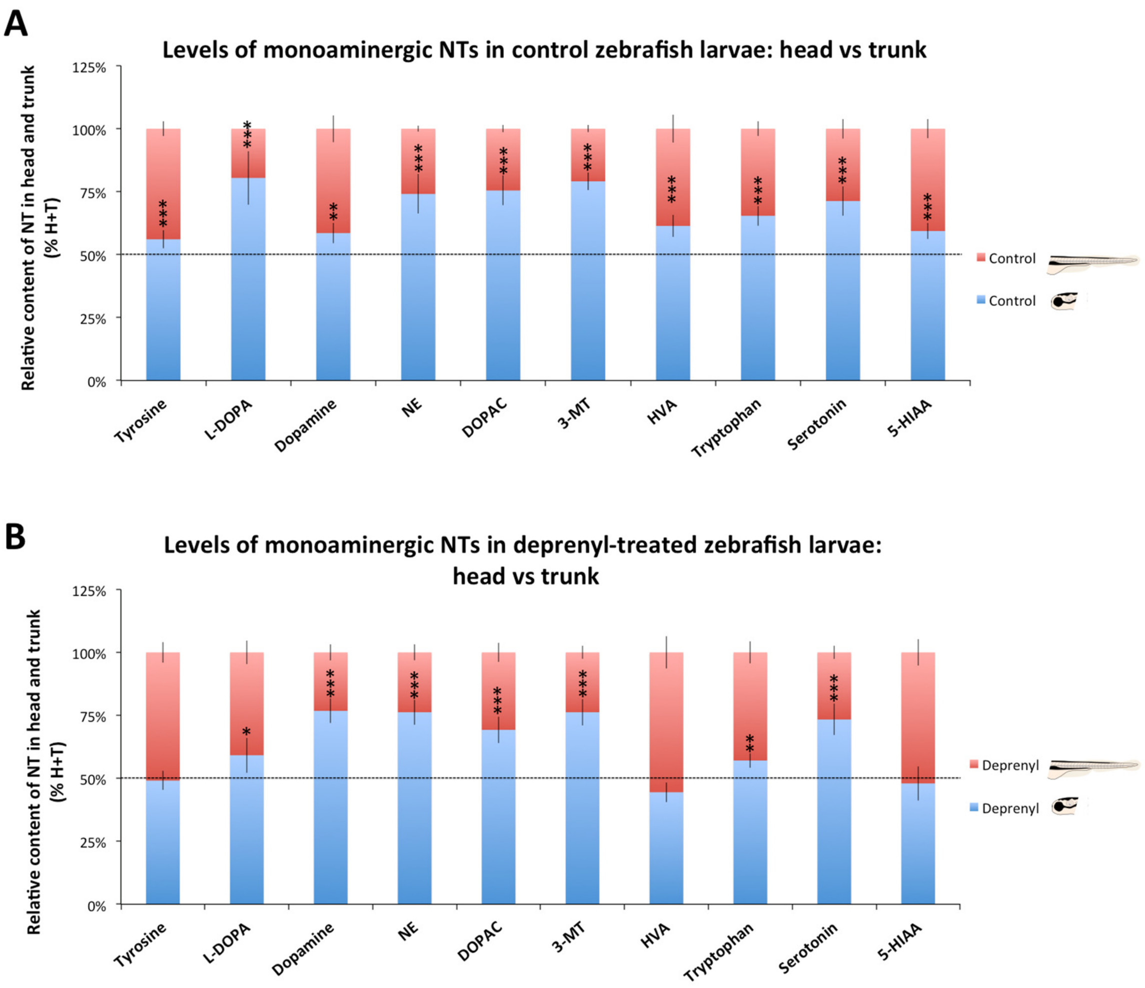

Figure S2. Levels of ten monoaminergic neurochemicals in the head and trunk of zebrafish larvae. (**A**) Distribution in the head and the trunk control 8 days post-fertilization (dpf) larvae; (**B**) Distribution in the head and the trunk of 8 dpf larvae exposed for 24h to 5 µM deprenyl, a monoamine-oxidase inhibitor, in the water. Data was normalized by larva (n = 8 pools) **P* < 0.05, ***P* < 0.01, ****P* < 0.001; Student's t-test; Data from 2 independent experiments.

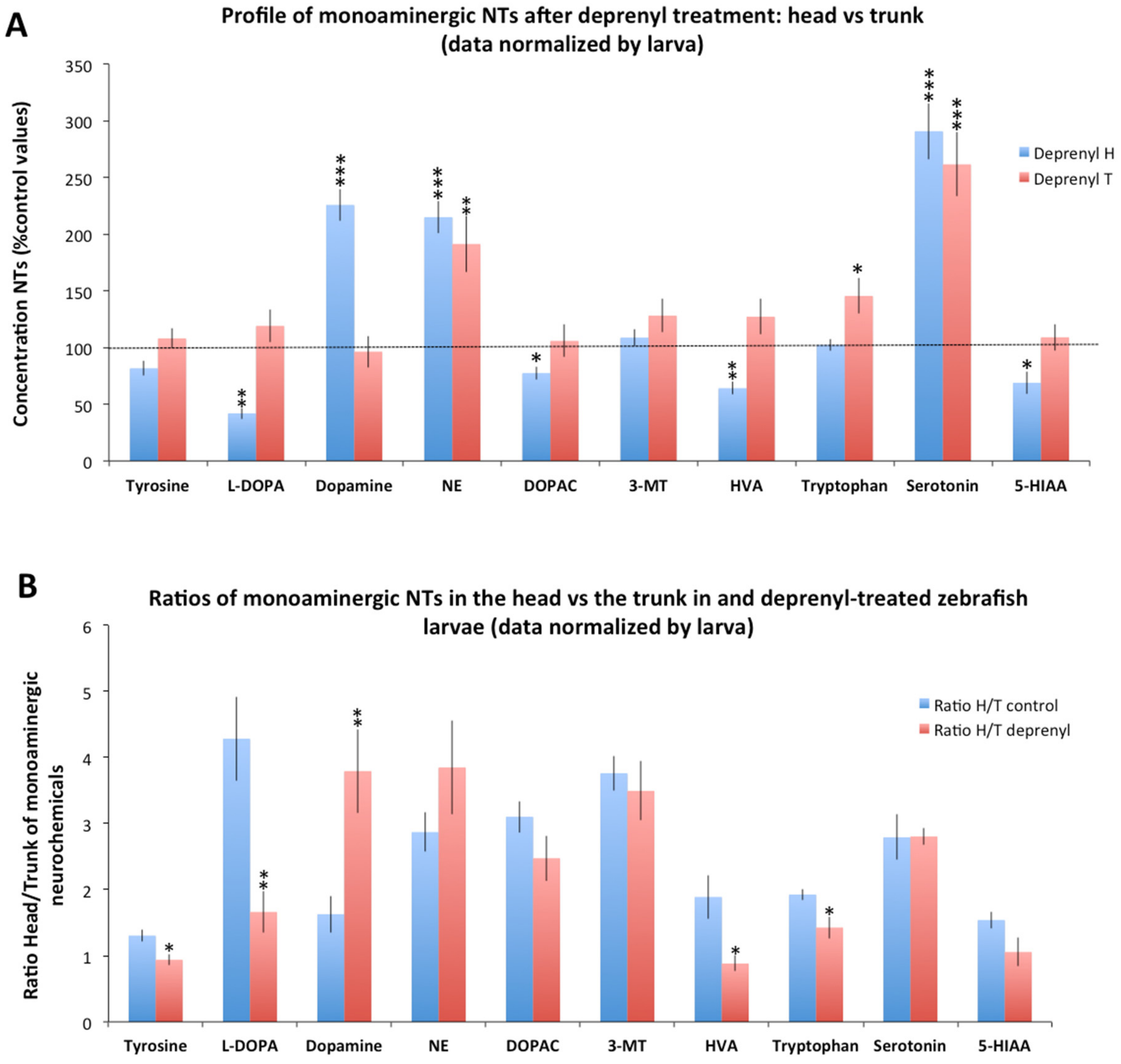

Figure S3. Distribution of ten monoaminergic neurochemicals between head and trunk of zebrafish larvae exposed to 5 µM deprenyl for 24h. (**A**) Concentration of monoaminergic neurochemicals in the head and trunk of larvae exposed to deprenyl, expressed as a percentage of the control values; (**B**) Ratio Head/Trunk for the ten selected monoaminergic neurochemicals in control and deprenyl-treated zebrafish larvae. Data was normalized by larva. Student's t-test (n = 8 pools); **P* < 0.05, ***P* < 0.01, ****P* < 0.001; Data from 2 independent experiments.

4. Supplementary Tables

Table S1. Quality parameters. Quality parameters obtained by LC-MS/MS for the 10 monoaminergic neurochemicals. F: slope, r^2: regression coefficient; IDL: instrumental detection limit; RSD: relative standard deviation; MDL: method detection limit; %R: recovery and %ME: matrix effect.

Monoamine neurochem*	F	r^2	IDL (pg)	Intra-day precision (RSD, %)	Inter-day precision (RSD, %)	Head			Trunk			Larvae		
						MDL (ng head^{-1})	%R ± RSD	%ME ± RSD	MDL (ng trunk^{-1})	%R ± RSD	%ME ± RSD	MDL (ng larvae^{-1})	%R ± RSD	%ME ± RSD
5-HT	0.6	0.9994	1.1	2.8	12.4	2.8	102±5	108±17	2.1	107±6	74±25	2.6	76±5	125±9
3-MT	1.9	0.9978	1.0	5.5	5.7	0.3	135±9	159±8	0.1	138±8	165±15	14.9	120±14	64±10
HVA	0.3	0.9983	33.3	4.1	8.0	44.3	95±16	106±2	34.9	94±19	116±12	54.5	67±19	109±17
DOPAC**	2.1e6	0.9972	2.7	5.5	7.7	1.1	111±9	114±10	1.3	100±14	105±14	8.0	112±15	59±8
Trp	2.2	0.9994	1.4	6.3	11.7	1.6	57±9	144±14	1.5	65±10	135±16	3.7	124±5	130±17
LD	2.4	0.9971	21.4	7.5	12.4	33.9	80±18	103±25	15.3	85±7	107±12	52.3	81±3	61±5
DA	8.3	0.9938	5.4	5.4	6.4	4.2	130±9	136±6	2.8	133±13	116±14	2.7	113±5	91±6
NE	1.2	0.9998	4.0	2.1	6.3	1.6	140±10	64±12	1.3	120±12	85±25	25.0	89±3	80±3
HIAA	3.3	0.9992	2.9	4.5	6.0	1.1	100±6	123±16	1.5	103±4	99±25	8.2	76±4	85±3
Tyr	1.4	0.9998	6.8	6.0	14.2	4.4	135±6	69±16	4.3	140±6	73±13	11.7	99±11	115±18

* The studied linearity ranges were from 0.005 to 2.5 ng μL^{-1}

**All neurochemicals were calibrated using internal standard, except for DOPAC (external calibration).

Table S2. Protein content in whole-body, heads and trunks of control and deprenyl-treated 8 days post-fertilization zebrafish larvae. The content is expressed in mg/mL in the pool.

	**Average**	**SE**	**N**	**Individuals per pool**
**Control:**				
Whole-body	0.1280	0.0090	4	5
Head	0.1939	0.0234	4	15
Trunk	0.1228	0.0052	4	15
**Deprenyl:**				
Whole-body	0.1396	0.0072	4	5
Head	0.2047	0.0086	4	15
Trunk	0.1553	0.0076	4	15
